# Functional Evaluation of Two Corneal Endothelial Cell-Based Therapies: Tissue-Engineered Construct and Cell Injection

**DOI:** 10.1038/s41598-019-42493-3

**Published:** 2019-04-15

**Authors:** Gary S. L. Peh, Hon Shing Ong, Khadijah Adnan, Heng-Pei Ang, Chan N. Lwin, Xin-Yi Seah, Shu-Jun Lin, Jodhbir S. Mehta

**Affiliations:** 1 Tissue Engineering and Stem Cell Group, Singapore Eye Research Institute, Singapore, Singapore; 2 Duke-NUS Graduate Medical School, Singapore, Singapore; 3 Singapore National Eye Centre, Singapore, Singapore; 4 School of Material Science and Engineering, Nanyang Technological University, Singapore, Singapore

## Abstract

Restoration of vision due to corneal blindness from corneal endothelial dysfunction can be achieved via a corneal transplantation. However, global shortage of donor tissues has driven the development cell-based therapeutics. With the capacity to propagate regulatory compliant human corneal endothelial cells (CEnCs), this study evaluated the functionality of propagated CEnCs delivered via tissue-engineered endothelial keratoplasty (TE-EK) or corneal endothelial cell injection (CE-CI) within a rabbit model of bullous keratopathy. For animals with TE-EK grafts, central corneal thickness (CCT) increased to >1000 μm post-operatively. Gradual thinning with improvements in corneal clarity was observed from week 1. CCT at week 3 was 484.3 ± 73.7 μm. In rabbits with CE-CI, corneal clarity was maintained throughout, and CCT at week 3 was 582.5 ± 171.5 μm. Control corneas remained significantly edematous throughout the study period compared to their respective experimental groups (*p* < 0.05). Characterization of excised corneas showed a monolayer with heterogeneously shaped CEnCs in both TE-EK and CE-CI groups. Immunohistochemistry demonstrated reactivity to anti-human specific nuclei antibody attributing corneal recovery to the functional human CEnCs. This study showed that regulatory compliant cell-based therapy for corneal endothelial dysfunction can be delivered by both TE-EK and CE-CI, and holds great promise as an alternative to traditional corneal transplantation.

## Introduction

Corneal diseases are the fourth leading cause of blindness in the world, after cataract, glaucoma, and age-related macular degeneration^1^. A significant sub-group of corneal diseases are due to a dysfunctional corneal endothelium (CE), with Fuchs’ corneal endothelial dystrophy (FED) and bullous keratopathy being the two commonest causes^2,3^. The human CE is the innermost single cell-layer of the cornea. It plays an important role in the maintenance of corneal hydration, keeping the cornea transparent, via a dynamic mechanism involving leaky barriers and active ionic pumps^4–6^. In damaged and diseased CE, any acute or accelerated loss of corneal endothelial cells (CEnCs) significant enough to cause a functional imbalance, will result in a leakier CE layer and weakened pump function, hindering regulation of corneal hydration^7–9^. Left untreated, the cornea becomes edematous over time, leading to a gradual loss of corneal transparency, and eventually, corneal blindness^10^.

The human CE is unable to regenerate within the eye^11,12^, due to its non-proliferative, quiescent nature^13,14^. The lack of regenerative capacity of the CE *in vivo* has since been attributed to a combination of factors including cell-cell contact-dependent inhibition, a lack of effective growth stimulation, and the presence of mitotic inhibitors such as transforming growth factor-β2 (TGF-β2) present within the aqueous humour^15–18^. However, it has been well described that CEnCs can be induced to proliferate *in vitro* when exposed to the appropriate culture conditions^19–21^. Various studies from our group and others have reported on the expansion of donor cornea derived CEnCs, with advancement made towards media formulation to increase the general growth dynamics and overall cellular yield of propagated CEnCs^21–26^. The most recent improvements made to the propagation of primary CEnCs have pushed the culture of these cells towards a regulatory compliant and well-defined system suitable for human clinical trials^27^.

The proven capacity to propagate CEnCs *in vitro* under good manufacturing practices (GMP) will undoubtedly increase the already growing interest in the potential of using expanded CEnCs for the treatment of CE dysfunction, where CEnCs isolated from one donor can potentially be propagated to benefit multiple recipients^28^. Without the advent of such disruptive, groundbreaking innovation in the form of cell-based therapies, the demand of corneal transplantation will only increase proportionally together with an aging global population^10^. This is due largely to the global shortage of suitable donor corneas available for individuals requiring corneal transplantation. Indeed, it was reported that in 2012 alone, whilst approximately 185,000 cases of corneal transplants were performed globally, the shortfall was significant with a worldwide demand that was conservatively estimated to be around 12.7 million^29^. This indicates that only approximately 1 in 70 of the global needs for corneal transplantation was met.

The ability to potentially treat multiple individuals through cellular therapy, using primary CEnCs propagated from a single cadaveric donor tissue, can only be realized with a capacity to deliver the expanded CEnCs into the eye, and a proven functionality of the delivered cells to maintain corneal deturgescence, keeping the cornea clear. Currently, two of the most plausible approaches for the delivery of expanded CEnCs described to date are: (i) tissue engineered endothelial keratoplasty (TE-EK)^27^, and (ii) direct corneal endothelial cell injection (CE-CI)^19,30^. For TE-EK, cultured CEnCs grown to confluence are dissociated into a single-cell suspension before being seeded at high density onto a thin biological scaffold carrier at 3,000 cells/mm^2^. The constructed TE-EK graft is left to stabilize over 5–7 days, before transplantation into the eye. The delivery of the TE-EK graft is based on existing EK surgeries, specifically Descemet’s stripping endothelial keratoplasty (DSEK) and Descemet’s stripping automated endothelial keratoplasty (DSAEK)^31,32^, and has recently been shown to successfully reverse corneal blindness in a rabbit model of bullous keratopathy^27^. The CE-CI approach involves fewer procedural steps. Cultivated CEnCs are firstly dissociated into a single-cell suspension, before being delivered by direct injection into the anterior chamber of the recipient. This is followed by at least three hours of posturing face-down to allow the injected CEnCs to settle by gravity and adhere onto the posterior corneal surface^19,33,34^. The conceptual simplicity of the minimally invasive CE-CI, relative to TE-EK, makes it an appealing approach. However, pre-clinical studies using CE-CI have reported conflicting functional outcomes^19,33–40^. Complete functional recovery of the CE could not be clearly demonstrated following the injection of CEnCs in a feline model^33^; whereas studies reported by Okumura and colleagues showed complete functional recovery of the CE in both the rabbit^39^ and non-human primate^40^ models of bullous keratopathy. It has since been reported, in a recently published clinical trial (UMIN000012534) that the injection of human CECs supplemented with ROCK inhibitor Y-27632 were able to repopulate and increase the CEC density of 11 patients with bullous keratopathy after 24 weeks^41^, potentially strengthening the push for a cell-injection approach.

With the development of cell-based therapies in mind, we have recently described the propagation of CEnCs, using a dual media culture system, formulated towards GMP-compliance^27^. For this study, the GMP-compliant CEnCs were expanded, characterized and assessed for functionality through two delivery approaches described above (TE-EK and CE-CI); using a rabbit model of bullous keratopathy. The availability of different delivery options based on two different mechanism will be important in future clinical application, where cell delivery may be dependent on a given pathological diagnosis^28^. More importantly, the differences in functional recovery of the edematous rabbit corneas receiving the two different preparations of CEnCs via the two modes of delivery were evaluated.

## Results

### Characterization of cultured CEnCs piror to transplantation

**Figure 1:**
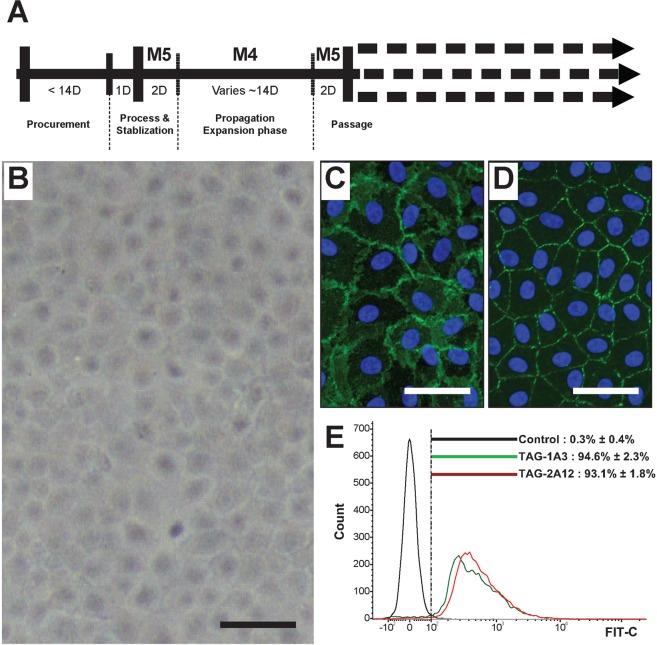
Culture and characterization of primary human CEnCs. (**A**) Schematic diagram depicting the dual media culture system used in this study, from the procurement stage through to the different phases of processing including isolation, stabilization, expansion and passaging. (**B**) Representative image of a confluent culture of primary human CEnCs at the third passage showing the homogeneous cellular morphology. Cultures of human CEnCs were characterized for expression of (**C**) Na^+^/K^+^-ATPase, and (**D**) ZO-1 by immunocytochemistry, as well as their expression of (**E**) Tag 1A3 - CD166, and Tag 2A12 - PRDX-6 and by flow cytometric analysis of live CEnCs. Scale bar: B 100 µm; C and D 50 µm.

Human CEnCs were isolated and propagated using a dual media approach as illustrated (Fig. 1A). For this study, CEnCs were cultured to either the second or third passage to facilitate cellular requirement whilst maintaining cellular homogeneity based on cellular morphology (Fig. 1B). Propagated CEnCs at both the second and third passage were found to express both function-associated ionic pumps Na^+^/K^+^-ATPase (Fig. 1C), and tight junction protein ZO-1 (Fig. 1D) by immunocytochemistry. Flow cytometric analysis of cultured CEnCs showed high expression of two cell-surface markers TAG-1A3^42^ (anti-CD166) at 94.6% ± 2.3% and TAG-2A12^42^ (anti-PRDX-6) at 93.1% ± 1.8% (Fig. 1E).

### Pre-operative assessment of rabbits following cataract extraction

**Figure 2:**
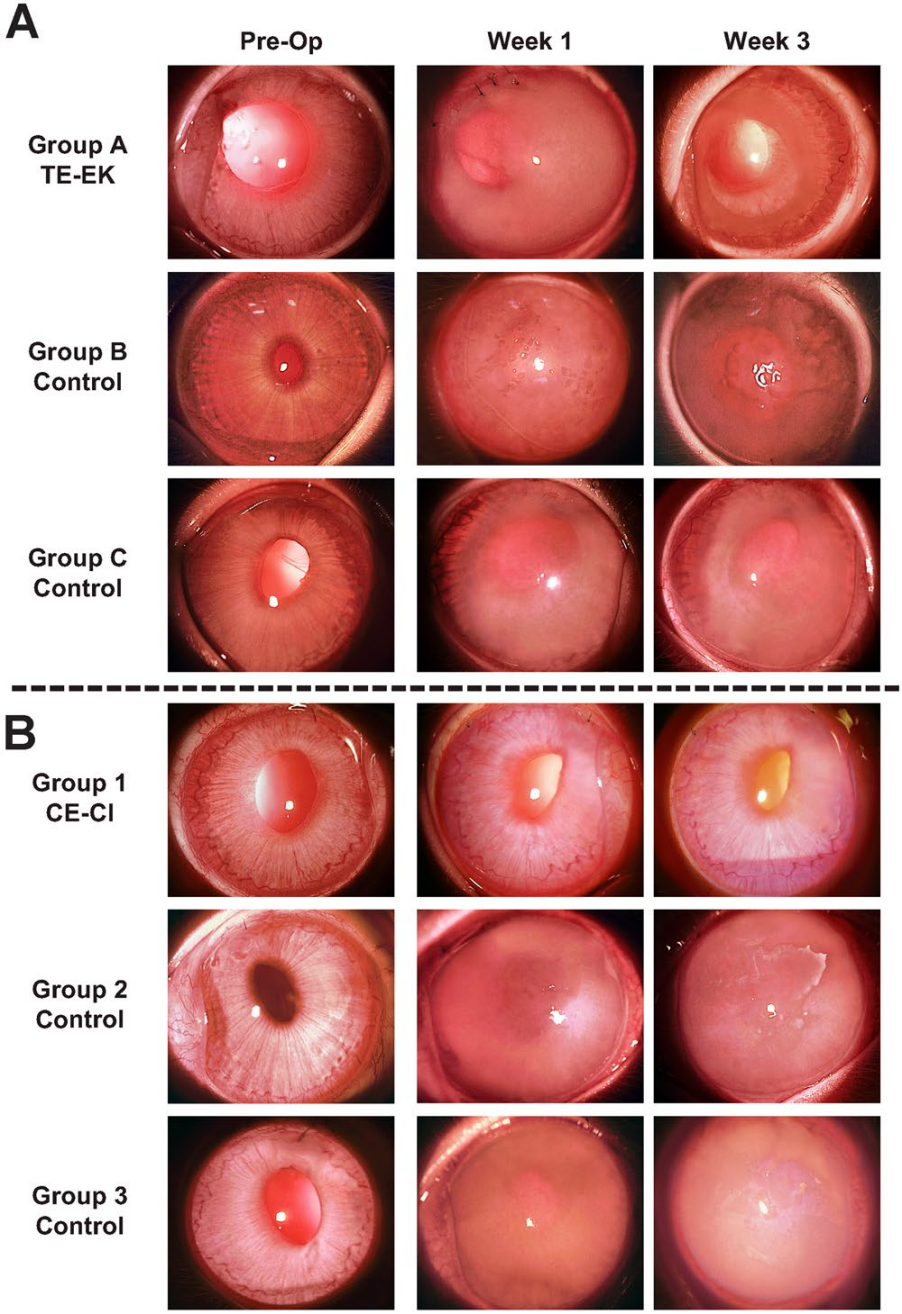
Slit-lamp images of rabbits from both experimental (TE-EK and CE-CI) groups. (**A**) Representative pre-operative, as well as week 1 and week 3 post-operative slit-lamp images of rabbits in Group A treatment group where DM of rabbits were removed before receiving TE-EK grafts; Group B Controls where the DM of rabbits were removed; and Group C Controls where DM of rabbits were removed and a blank carrier inserted. (**B**) Representative pre-operative, as well as week 1 and week 3 post-operative slit-lamp images of rabbits in Group 1 rabbits where DM of rabbits were scrapped before receiving CE-CI; Group 2 Controls where the DM of rabbits were removed before receiving CE-CI; and Group 3 Controls where the DM of rabbits were scrapped with no cells injected.

All rabbits were assessed one week after lens extraction just before TE-EK or CE-CI surgeries (Fig. 2). The corneas of all rabbits were clear with no visible epithelial defects, opacities, or vascularisation, and no intraocular inflammation was observed. The mean CCT of rabbits in the TE-EK treatment and control groups was 392.4 μm ± 27.5 μm in the Group A TE-EK (n = 3), 394.2 μm ± 30.7 μm in Group B control (n = 3), and 400.6 μm ± 29.1 μm in Group C control (n = 3), with no significant difference (p = 0.708) in pre-operative CCT among groups. For rabbits within the CE-CI and control groups, mean CCT of rabbits were 357.1 μm ± 60.7 μm in Group 1 (n = 5), 373.9 μm ± 24.0 μm in Group 2 (n = 3), and 406.8 μm ± 47.0 μm in Group 3 (n = 3). There were no significant difference (p = 0.627) among the groups.

### Post-operative clinical outcomes in rabbits

#### Corneal transparency

TE-EK and controls: In the eyes of the rabbits in Group A TE-EK, an area of corneal clarity, which corresponded to the size of the graft was observed by week 1, and continued improving up to week 3. The peripheral region of the corneas, outside the area of the transplanted graft where DM was stripped and removed, remained hazy throughout the study period. For the eyes of rabbits in both Group B and Group C controls, the corneas remained hazy throughout the post-operative period (Fig. 2). No significant IOP elevation was noted in any of the post-operative eyes.

CE-CI and controls: Following CE-CI procedure, there were signs of intraocular inflammation with mild flare observed in the anterior chamber. These resolved within one week after surgery. The corneas of rabbits receiving the injection of CEnCs in Group 1 CE-CI were progressively clearer throughout the follow-up period, and this corneal clarity was maintained throughout the length of the study. The corneas of rabbits in Group 2 and Group 3 controls remained hazy throughout the post-operative period (Fig. 2). No significant IOP elevation was noted in any of the post-operative eyes.

#### Central Corneal thickness

**Figure 3:**
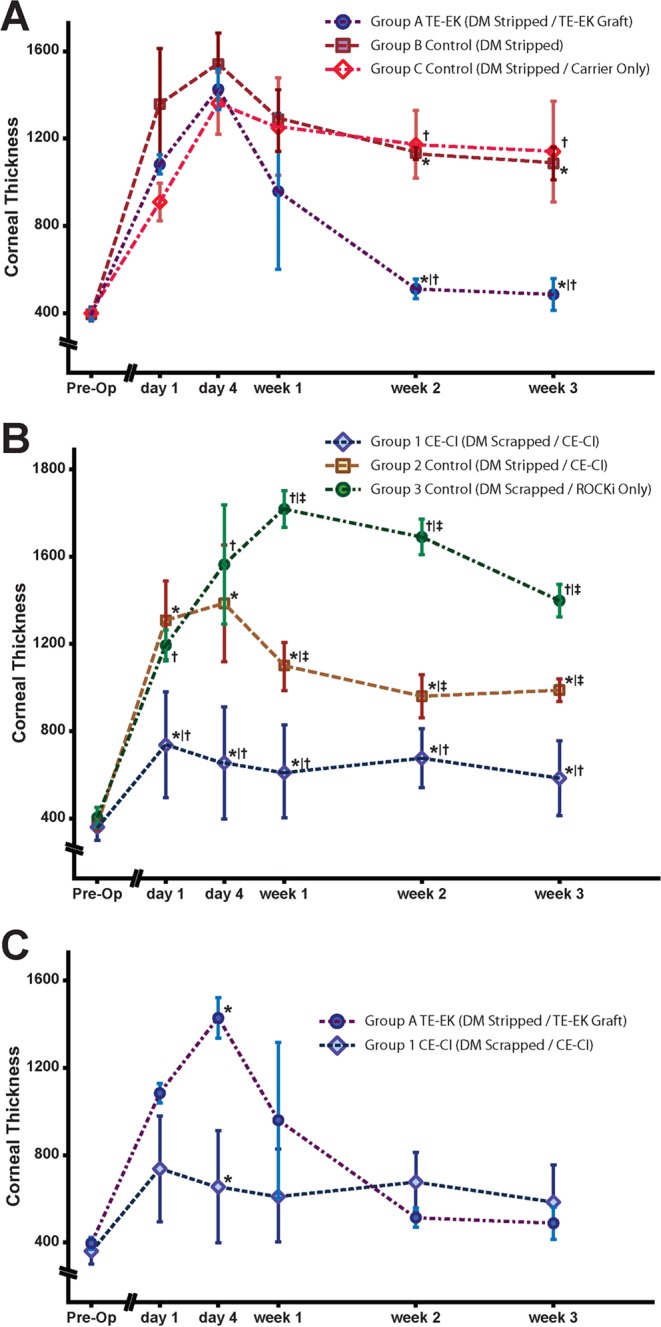
Comparative corneal thickness of rabbits receiving TE-EK grafts and CE-CI. Corneal thickness of rabbits were collected pre-operatively, day 1, day 4, week 1, week 2, and week 3. Time-points were collected and graphs were plotted to scale. (**A**) Graph summarizing the corneal thickness of rabbits in Group A TE-EK treatment group against its respective 2 control groups. (**B**) Graph summarizing the corneal thickness of rabbits of Group 1 CE-CI group and its respective 2 control groups. (**C**) Comparison of corneal thickness of rabbits in Group A receiving TE-EK grafts and Group 1 CE-CI receiving an injection of primary CEnCs over the 3 weeks study period.

TE-EK and controls: Following the transplantation of the tissue-engineered grafts into rabbits in treatment Group A - TE-EK, corneal thickness increased over the first 4 days to above 1,000 µm. Thereafter, the corneas of these rabbits gradually thinned by the second week, and the mean corneal thickness at week 3 was 484.3 µm ± 73.6 µm. Similar to the rabbits in Group A TE-EK, the corneal thickness of rabbits in Groups B and Group C controls increased gradually over the first 4 days. However, the corneas of rabbits in both control groups remained thick at over 1,000 µm throughout the 3 weeks observation period. By week 2, the mean corneal thickness of rabbits in Group A TE-EK was 509.9 µm ± 44.5 µm, and this was significantly thinner when compared to the corneas of rabbits from Group B control 1134.4 µm ± 28.4 µm (*p < 0.05) and Group C control 1173.3 µm ± 155.2 µm (†p < 0.05). As stated, at week 3, the corneal thickness of rabbits in Group A TE-EK reduced to 484.3 µm ± 73.7 µm, whereas the corneas remained significantly thicker at 1087.2 µm ± 74.7 µm for Group B control (*p < 0.05; Fig. 3A), and 1140.6 µm ± 231.0 µm for Group C control (†p < 0.05; Fig. 3A). There was no observable corneal recovery for either control group (Fig. 3).

CE-CI and controls: Following CE-CI, the mean corneal thickness of rabbits in Group 1 CE-CI increased to 735.6 μm ± 243.0 μm on day 1. It should be noted that the 3-hours recovery period was critical to the surgical outcome in terms of corneal thickness on the following day. For example, two extreme outcomes were observed within Group 1 CE-CI at day 1 where two CE-CI procedures were performed on two rabbits, one after another. With all variables kept the same, down to the batch of cultured CEnCs used, one of the rabbits was seen to have moved its head briefly during the recovery period. As a result, the corneal thickness at day 1 for that rabbit was 831.3 μm ± 40.0 μm, whereas the corneal thickness of the rabbit that remained still throughout the recovery period was 332.0 μm ± 10.5 μm. Generally, subsequent mean corneal thickness of Group 1 CE-CI rabbits were relatively lower, and were observed to be 652.9 μm ± 257.3 μm at day 4, 613.4 μm ± 213.6 μm at week 1, 674.5 μm ± 136.3 μm at week 2, and 582.5 μm ± 171.5 μm at week 3. Compared to the corneal thickness of Group 1 CE-CI rabbits, the corneas of rabbits in Group 2 (*p < 0.05; Fig. 3B) and Group 3 (†p < 0.05; Fig. 3B) controls were significantly thicker from day 1 onwards, and throughout the duration of the study. Interestingly, the corneal thickness of Group 2 controls was significantly thinner than Group 3 controls (‡p < 0.05; Fig. 3B) at week 1, week 2, and week 3 respectively.

TE-EK versus CE-CI: Between the two treatment groups, Group A TE-EK and Group 1 CE-CI, the corneas of Group A TE-EK rabbits were significantly thicker at 1457.5 µm ± 99.0 µm on day 4, compared to corneas of Group 1 CE-CI rabbits with mean corneal thickness of 652.6 μm ± 257.4 μm (*p < 0.05; Fig. 3C, Supplementary Figure S1). Thereafter, by post-operative week 1, the corneas of rabbits in Group A TE-EK thinned down gradually, but although still thicker than the corneas of Group 1 CE-CI rabbits, the differences were insignificant. In subsequent weeks, the corneas of rabbits in Group A TE-EK became generally thinner than that of Group 1 CE-CI rabbits, but no statistical significance in corneal thickness between the 2 groups were observed.

#### *In vivo* confocal microscopy and endothelial cell density of treatment groups

TE-EK experiments: Due to the initial thickening and poor corneal clarity of rabbits receiving the TE-EK grafts in Group A TE-EK, *in vivo* confocal images were unobtainable until the corneas thinned down at week 2, where a relatively confluent mosaic layer of polygonal CEnCs was observed. The mean endothelial cell density at week 3 was assessed to be approximately 1248 ± 64 cells/mm^2^, with a cell circularity of 0.86 ± 0.04 (Supplementary Figure S1).

CE-CI experiments: The corneas of rabbits receiving CE-CI remained relatively thin throughout the course of the study for the periodic capture of *in vivo* confocal images, which revealed a confluent layer of polygonal CEnCs in a mosaic pattern from post-operative day 4 onwards. This was maintained throughout the follow-up period. The mean endothelial cell densities assessed at day 4 was 1986 ± 266 cells/mm^2^ and at week 3 was 1409 ± 128 cells/mm^2^, with a cell circularity of 0.84 ± 0.06 (Supplementary Figure S1).

### Characterization of excised corneas from TE-EK and CE-CI procedures

**Figure 4:**
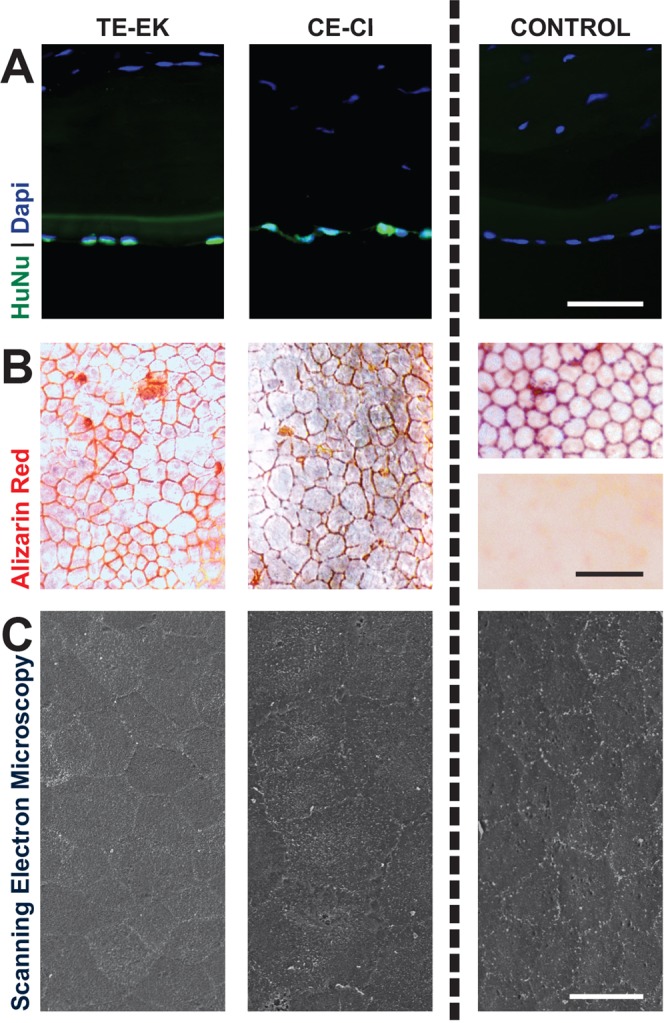
Characterization of excised corneas. (**A**) Immuno-staining of human-specific nuclei antibody were performed on sections of excised corneas of rabbits receiving TE-EK grafts or CE-CI, as well as on sections of rabbit corneas as control. (**B**) Flat-mount Trypan Blue and Alizarin Red staining of rabbits receiving TE-EK grafts or CE-CI. Controls included the staining the rabbit naïve corneal endothelium (Top) and bare rabbit stroma where the DM was stripped (Bottom). (**C**) Representative SEM images of excised corneas from the experimental TE-EK, CE-CI and control groups.

In order to show that the rabbit’s corneal recovery were due to the functioning human CEnCs on the TE-EK grafts and the injected CEnCs for the respective Group A TE-EK and Group 1 CE-CI procedures, representative CEnCs from the experimental specimens were found to be reactive to an anti-human specific nuclei antibody. In contrast, tissues obtained from control rabbit corneas were not reactive (Fig. 4A). Flat-mounted histological analysis of Trypan Blue / Alizarin Red staining of excised corneas from representative TE-EK and CE-CI procedures showed similar cellular layers with heterogeneously shaped cells that were irregularly polygonal (Fig. 4B). Compared to control rabbit CE, the cells were homogenous and regular in shape (Fig. 4B, Control – top panel); whereas in control rabbits with bare corneal stroma, no cells were detected (Fig. 4B, Control – bottom panel). Representative SEM images of excised corneas from both TE-EK and CE-CI procedures showed the irregular shapes of human CEnCs on the TE-EK graft and the established CE of the injected human CEnCs respectively. This is in contrast to the homogenous mosaic pattern of the rabbit’s own corneal endothelium (Fig. 4C).

## Discussion

To successfully develop corneal endothelial cell-based therapeutics towards alleviating global dependency on donor corneas for CE-associated corneal transplantation is entirely dependent on two critical components: (1) the ability to propagate primary human CEnCs within a clean facility, with regulatory approval for clinical use; and (2) the capacity to deliver the expanded CEnCs using clinically translatable methodologies with robust consistency, as well as validated functionality in terms of sustaining corneal deturgescence and maintaining corneal transparency. Building upon our previous report on the refinement of the dual media culture system for the propagation of functional primary human CEnCs towards regulatory compliance, we now show in this study that the expanded CEnCs can be delivered into the AC via both the conventional tissue-engineered carrier-based ‘TE-EK’ approach as previously described^27^, as well as by the cell-injection ‘CE-CI’ approach. More importantly, we were able to show that CEnCs delivered by both approaches were able to reverse corneal blindness in a rabbit model of bullous keratopathy as shown by the recovery in corneal thickness in both treatment groups.

For the TE-EK approach, its main concept lies in the generation of a tissue-engineered graft material similar to that of ultrathin DSAEK graft^27^, where propagated human CEnCs are seeded at a specified physiological density onto a thin DM-intact stromal lenticule. More importantly, the insertion of TE-EK grafts, was based on current DSAEK insertion technique^43^, demonstrated the adaptability and the clinical translatability of TE-EK^27^. However, the current approach in our generation of TE-EK graft material requires the use of an additional donor cornea with an intact DM. Although such donor tissue can be derived from suitable older donor tissues that were initially retrieved for clinical use but subsequently rejected due to a myriad of potential reasons and will otherwise be discarded^10^, overall cost-effectiveness of TE-EK^44^ will be impacted. Indeed, the need of an additional donor tissue will inevitably incur additional costs including mandatory screenings of infectious diseases, as well as the procurement logistics involved. Hence, future studies driving the development of TE-EK grafts towards a more sustainable approach must involve the development of suitable biosynthetic carrier such as gelatin methacryloyl based hydrogels^45^.

Alternatively, another approach of delivering cultured CEnCs into the anterior chamber is through intracameral cell injection, which has been described in earlier reports in various animal models (rabbit, feline, primate) of bullous keratopathy^33,39,40^, and more recently in a breakthrough clinical trial by Kinoshita and colleagues^41^. Injection of the CEnCs into the anterior chamber is a conceptually simpler and is a minimally invasive procedure compared to TE-EK. However *in vivo* studies using the cell injection approach has not been successfully replicated by other independent research groups where outcomes have been conflicting^33,39,40^. In this current study, our attempt to deliver propagated CEnCs by intracameral cell injection resulted in positive functional recovery within a similar rabbit model of bullous keratopathy. Here, following removal of the rabbits’ native CEnCs via scraping of the DM, eyes that received the injected cultured CEnCs maintained corneal clarity throughout the study period, and were significantly thinner compared to the two control groups, indicating a functional corneal endothelium. In accordance with previously reported studies of cell injection, elevation of intraocular pressure was not detected, suggesting that injected CEnCs did not obstruct the trabecular meshwork^33,39^.

Interestingly, the functional recovery profile of the corneas of rabbits receiving CE-CI was different to that of rabbits receiving TE-EK graft, which first became edematous for at least 1 week following surgery before signs of functional recovery were observed (Fig. 3C). Significant reduction in corneal thickness was only detected two weeks following TE-EK surgery (Figs 2, 3, and Supplementary Figure S1). These observations indicated that a minimally invasive procedure such as CE-CI may potentially result in faster recovery. However, it should be noted that, due to the sensitivity of CE-CI, several precedents must be maintained to achieve a positive therapeutic outcome. First, any form of movement during the three hours of mandatory inertia will disrupt the adherence of the injected CEnCs, and affect corneal thickness on the following day. Hence, the subject must remain relatively still following CE-CI for the initial three hours. Second, the final resting position of the rabbit’s eye was found to be absolutely crucial. For example, in one rabbit, improper positioning resulted in the accumulation of injected CEnCs over one side of the posterior corneal surface, resulting in a thinner cornea (590.3 μm ± 44.8 μm) on the side where the CEnCs had accumulated, and a much thicker cornea (1086.7 μm ± 216.4 μm) on the contra-lateral side (Supplementary Figure S2). Third, and perhaps the most important findings in this study was the presence of an intact DM, and this is paramount to the success of CE-CI. We found in Group 2 Control where the DM was removed, injected CEnCs were unable to improve corneal transparency or reduce corneal thickness throughout the study period. This is in contrast to a recent study by Okumura and colleagues which suggested that it was possible for injected rabbit’s corneal endothelial cells to adhere onto the corneal stroma following descemetorhexis^46^. Subsequent characterization and histological analysis of corneas retrieved from Group 2 Control showed a barren stroma with no evidence of a monolayer formation or any cellular attachment. These observations were consistent with a recently reported finding showing the importance of the DM in facilitating corneal endothelial wound healing and maintaining normal cellular phenotype^47^. As such, this has led other investigators towards the development of biosynthetic DM-like basement membranes for use in future corneal endothelial cell-based therapies^48^.

In this study, the removal of the whole rabbit CE by scrapping, in preparation of CE-CI meant that an injection of a large number of CEnCs (6.0 × 10^5^ cells) was required. However, from a clinical standpoint, if CE-CI were to be translated into clinical practice, a smaller number of CEnCs will be required, based on the current approach for treating corneal endothelial failure, where the CEnCs in the central 6 to 8 mm of the diseased cornea is removed. In addition to this, the availability of an additional surgical option to deliver CEnCs may be useful as each may be applied in different clinical situations, based on the underlying pathophysiology of the corneal endothelial disease, as well as on the condition of the DM. For example, in early stages of pseudophakic bullous keratopathy, where the DM is not severely damaged or scarred, the removal of CEnCs through DM scraping followed by replacement of CEnCs through cell injection may be the procedure of choice. Conversely, in late stages of bullous keratopathy where scarring of DM has occurred, the damaged DM will have to be removed. The procedure of choice for these patients would then be TE-EK, as CE-CI may not work as well in the absence of a DM^47^. Similarly in FED, TE-EK will be required instead of CE-CI as the deposition of extracellular matrix excrescences known as guttae on the DM are known to adversely impact visual function^49,50^. Furthermore, we have previously demonstrated in a bioengineered topographic model, that simulated densely packed guttata, similar to those observed in severe FED affected the formation of a CEnC monolayer^51^.

In conclusion, we have demonstrated succinctly in this study: the capacity to isolate and propagate GMP-compliant CEnCs using a dual media approach, as well as for the first time, the capacity to deliver the propagated GMP-compliant CEnCs using both the TE-EK and CE-CI approaches, with evidence of cellular functionality in reversing corneal blindness in a model of rabbit bullous keratopathy. It should be noted here that the required regulatory approval and compliance of propagated primary CEnCs will be based on the regulatory framework that is put in place, under the purview of local or regional regulators where the cellular therapy will take place. For example, in USA, that will be the Food and Drug Administration (FDA); and in UK, the Medicines and Healthcare products Regulatory Agency (MHRA). Here in Singapore, in order to satisfy the requirement as defined by Health Science Authority (HSA), the regulators within Singapore, and specifically, the Health Products Regulation Group of HSA, the propagated primary CEnCs have to undergo stringent scrutiny and thorough documentation at each step of the entirety of the process, from procurement of donor corneas required for cellular propagation, and the process of corneal endothelial cell expansion. Due partly to the stringent selection criteria of the donor corneas (see Table 1) procured for this study, and partly to the robustness of the dual media approach of corneal endothelial cell expansion, all isolated CEnCs propagated to the second passage were relatively homogeneous and were used in the preparation of TE-EK graft or for CE-CI in the present study. However, if propagated CEnCs were severely heterogeneous, or showed signs of endothelial-to-mesenchymal transition, they will be deemed unsuitable for cellular therapy, withdrawn from the procedure, and replaced. Next, and specifically to the generation of the TE-EK graft, the procurement of the donor corneas required as scaffold carrier; the tissue engineering of the graft, as well as product release testing, right down to the transport of the prepared TE-EK graft from the GMP facility to the operating theatre were vetted and approved by HSA. Finally, to be able to release the prepared tissue-engineered graft from the GMP facility, several criteria has to be met. These include a series of sterility, mycoplasma and endo-toxin tests, as well as basic cellular characterization by immunocytochemistry for function-associated markers Na^+^/K^+^-ATPase and ZO-1, as well as by flow cytometry for cell surface markers Tag 1A3 – CD166 and Tag 2A12 – PRDX-6.

**Table 1:** Summary of donor information.

Serial Number	Sex	Age	Days to Culture	Cell Count (OS/OD)	Cause of Death
1	M	28	8	3106/3125	Overdose
2	F	3	9	4082/3968	Drowning
3	M	9	11	3096/3247	Anoxia
4	F	29	8	2591/2392	Multi-Vehicle Accident
5	F	19	12	3175/2890	Craniocervical Dislocation
6	F	24	8	2950/2865	SI-GSW-Head
7	F	17	11	3571/3472	Hanging
8	F	15	12	2809/2985	Multiple Blunt Force Injuries
9	M	13	15	3175/3058	Anoxia
10	F	35	5	2899/2941	Overdose
11	F	19	7	2681/2882	Acute Cardiac Arrest
12	F	11	10	3040/2907	Drowning
13	F	4	8	2717/3623	Anoxic Encephalopathy
14	F	23	8	2601/2398	Multi-Vehicle Accident
15	M	25	12	2959/3040	Multi-Vehicle Accident
16	F	2	12	4000/4016	Embryonal tumor with multilayered rosettes
17	M	35	9	2907/3012	Multi-Vehicle Accident
18	M	36	6	2915/3289	Multi-Vehicle Accident
19	F	19	12	3021 (OD)	Hypoxic Encephalopathy
20	M	58	DNC	N/A	Multi-System Organ Failure
21	M	69	DNC	N/A	Severe Malnutrition
22	F	63	DNC	N/A	Intracerebral Hemorrhage
23	M	53	DNC	N/A	Hepatic Encephalopathy
24	M	66	DNC	N/A	Myocardial Infarction

For corneal endothelial cell culture (serial number 1 to 19), donor age ranged from 2 year old to 36 year old with a median age of 19 year old. Days taken from death of donor to the initiation of corneal endothelial cell culture ranged from 5 days to 15 days with a median of 9 days. Serial numbers 1 to 18 were paired donor corneas, whereas serial number 19 was a single donor cornea. For the generation of tissue-engineered grafts (serial number 20 to 24), donor age ranged from 53 year old to 69 year old.

Subsequent clinical trials with adequate follow-up will be required to further evaluate both long-term safety and efficacy of the two delivery approaches. In fact, we have recently obtained the approval from Health Sciences Authority, Singapore (Clinical Trial Certificate: CTC1800013) to initiate a first-in-man clinical trial for TE-EK for the treatment of FED as well as all forms of pseudophakic or aphakic bullous keratopathy. With the rapid advancement in this field of research, cell-based therapies for the treatment of CE dysfunction indeed hold great promises, and their translation into clinical practice as an alternative to traditional corneal transplantation lies on the horizon.

## Methods

### Materials

Ham’s F12, Medium 199, Human Endothelial-SFM, Dulbecco’s Phosphate-Buffered Saline (PBS), TrypLE^TM^ Select (TS), gentamicin, amphotericin B, penicillin and streptomycin were purchased from Life Technologies (California, USA). Dimethyl sulfoxide (DMSO), trypan blue (0.4%), alizarin red, paraformaldehyde (PFA), and Collagen IV from human placenta were purchased from Sigma (Missouri, USA). Human recombinant basic fibroblast growth factor (HrFGF), and Rho-associated, coiled-coil protein kinase inhibitor (ROCKi), Y-27632, was purchased from Miltenyi Biotec (Bergisch Gladbach, Germany). Liberase TH was purchased from Roche (Mannhein, Germany). EquaFetal® was from Atlas Biologicals (Colorado, USA). Insulin/Transferrin/Selenium (ITS) was purchased from Corning (New York, USA), and ascorbic acid from Avantor (Pennsylvania USA).

### Research-grade human corneoscleral tissues

This study was approved by Singhealth centralized institutional review board (Ref: 2016/2839). All research-grade human cadaver corneal tissues were procured from either Lions Eye Institute for Transplant and Research (Florida, USA) or Miracles in Sights (North Carolina, USA), with informed consent from the next of kin. All research performed with human derived tissue was carried out in accordance to the principles outlined in the Declaration of Helsinki. All corneo-scleral donor tissues were preserved and transported in Optisol-GS (Bausch & Lomb, New York, USA) at 4 °C until they were processed.

### Cell isolation and cell culture

For isolation and culture of CEnCs, a total of 37 donor corneal tissues (18 pairs and 1 single) were procured for this study. Donors’ age ranged from 2 to 36 years of age (Table 1) with endothelial cell count of at least 2,200 cells per mm^2^. Propagation of CEnCs for this study was achieved using a dual media culture system^23^, refined towards GMP compliance as described^27^. Briefly, CEnCs were isolated using a two-step enzymatic treatment to first release the CEnCs from the DM (up to 4 hours), followed by a secondary brief 5-minute dissociation step to further dissociate the cellular clusters into smaller clumps. Isolated cells were briefly rinsed twice before being seeded onto pre-coated collagen culture vessels at a seeding density of 1.0 × 10^4^ cells per cm^2^ and established in a cornea endothelial maintenance/stabilization medium (M5-Endo; Human Endothelial-SFM supplemented with 5% serum) overnight. Subsequently, CEnCs were cultured in the proliferative medium (M4-F99; Ham’s F12/M199, 5% serum, 20 μg/ml ascorbic acid, 1x ITS, and 10 ng/ml HrFGF) to promote the proliferation of the attached CEnCs. Once the CEnCs reached 80% to 90% confluence, cells were re-exposed to M5-Endo for at least two days before being sub-cultured via single-cell dissociation using TS. Dissociated CEnCs were re-plated at a seeding density of at least 1.0 × 10^4^ cells per cm^2^ on pre-coated collagen surfaces for further expansion; or for subsequent studies, which included (1) seeding at higher densities for characterization; (2) preparation of the TE-EK graft; and (3) for direct CE-CI (see below). All cultures were incubated in a humidified atmosphere at 37 °C and 5% CO_2_.

For cellular characterization, CEnCs were cultured up to the second or third passage. Cellular morphology and homogeneity were assessed using phase contrast microscopy (Nikon DS-Fi1 digital camera, Tokyo, Japan). The expressions of function-associated ionic pumps Na^+^/K^+^-ATPase (5 µg/mL; Santa Cruz Biotechnology, Texas, USA) and tight junction protein ZO-1 (2 µg/mL; Thermo Fisher, Massachusetts, USA) were evaluated by immunocytochemistry. Finally, the expression of two cell-surface markers TAG-1A3^42^ (anti-CD166) and TAG-2A12^42^ (anti-PRDX-6) was assessed by flow cytometry using a FACS Verse flow cytometer (Becton Dickinson, New Jersey, USA).

### Preparation of tissue-engineered corneal endothelial graft

The generation of the tissue-engineered corneal endothelial grafts used in this study has been previously described^27^. Briefly, 5 pairs of donor corneas between 53 to 69 years of age (Table 1) that were deemed unsuitable for both transplantation as well as cellular expansion of CEnCs, were prepared as construct carriers for cell and tissue engineering of the TE-EK graft material. Selection criteria for these donor corneas were older than 50 years old, and/or greater than 14 days of preservation; and/or have endothelial cell counts under 2,000 cells/mm^2^, with an intact and undamaged DM. Laser dissection of 100 μm thick human corneal stromal lenticules with DM intact (diameter ≥8.0 mm) was performed using a femtosecond laser system (LDV, Ziemer, Port, Switzerland) as described^52^. Following laser dissection, the DM/stroma lenticule was gently separated, carefully transferred into a 4-well plate, and left fully submerged in PBS. Subsequently, the DM/stroma lenticule were denuded by three freeze/thaw cycles, and stored at −20 °C until used. At least a week before the scheduled transplantation study, the denuded frozen DM/stroma lenticules were thawed out and prepared as a 6.0 mm circular disc using a 6.0 mm corneal trephine blade (Solan Medtronics, Florida, USA), and left in M5-Endo medium overnight to ascertain general sterility. Hereafter, primary human CEnCs at the first or second passage were dissociated and seeded onto the 6.0 mm DM/stroma lenticules at a physiological density of 3,000 cells per mm^2^ (approximately 8.5 × 10^4^ cells), and maintained in M5-Endo medium within a humidified atmosphere at 37 °C and 5% CO_2_, with medium refreshed every 2 days for approximately 5 to 7 days until the day of surgery.

### Preparation of cultured human CEnCs for CE-CI surgery

Human CEnCs expanded to the second passage were dissociated, and re-suspended at a concentration of 6.0 × 10^5^ cells in 150 μl of M5-Endo containing ROCKi Y-27632 within a 1.0 ml syringe attached to a 30-gauge needle. It should be noted that passing cultured CEnCs through a 30-gauge needle was not detrimental to the overall cellular viability of the cells as assessed separately using a Fluorescein isothiocyanate (FIT-C) Annexin V apoptosis detection kit with Propidium Iodide through flow cytometry (Supplementary Figure S3).

### Animal surgeries

New Zealand White rabbits (n = 20) were used for this study and all TE-EK surgeries and CE-CI procedures were performed by JSM. Their use, care and treatment strictly adhered to the regulation of the ARVO statement for the Use of Animals in Ophthalmic and Vision Research, and all experimental procedures were approved by the Institutional Animal Care and Use Committee of SingHealth, Singapore (Ref: 2017/SHS/1292). All surgical procedures and follow-up evaluations were performed under general anesthesia achieved by intramuscular injections of 5 mg/kg xylazine hydrochloride (Troy Laboratories, New South Wales, Australia) and 50 mg/kg ketamine hydrochloride (Parnell Laboratories, New South Wales, Australia), along with topical application of lignocaine hydrochloride 1% (Pfizer Laboratories, New York, USA).

#### Lens extraction surgery

The crystalline lenses of rabbits were extracted using a standard phacoemulsification technique as described^53^. To achieve mydriasis, tropicamide 1% (Alcon Laboratories, Texas, USA) and phenylephrine hydrochloride 2.5% (Alcon Laboratories) eye drops were administered approximately 30 minutes before surgery. A clear corneal incision was made with a 2.8 mm disposable keratome. A 5.0 mm diameter continuous curvilinear capsulotomy of the anterior capsule was created under viscoelastic material (Viscoat; Alcon Laboratories) instilled into the anterior chamber. Hydro-dissection was performed using a 27-gauge cannula. The lens was then aspirated and removed with a standard phacoemulsification procedure using the White Star phacoemulsification system (Abbott Medical Optics, California, USA). Subsequently, the corneal incision was sutured with 10/0 nylon suture and the rabbits were left aphakic with an intact posterior capsule for at least one week before the cell-based procedures.

#### TE-EK surgery

The insertion of TE-EK graft was carried out using the EndoGlide insertion technique^43^ with modifications made to facilitate the graft insertion into the anterior chamber of the rabbit as previously described^27^. For the TE-EK study, rabbits in the treatment group where transplanted with the TE-EK grafts (n = 3; Group A TE-EK). The two groups of control for this part of the study included rabbits that had DM stripped and removed without receiving a graft (n = 3; Group B control), and those that received an empty DM/stroma lenticule without any seeded human CEnCs (n = 3; Group C control).

#### CE-CI surgery

For the CE-CI study, the DM of the rabbits in the treatment group were left intact and these rabbits received a single injection of 6.0 × 10^5^ human CEnCs following the scraping of the DM to remove the rabbit’s native CEnCs (n = 5; Group 1 CE-CI). Similar to the TE-EK study, there were two control groups, one which involved the removal of rabbits’ DM before the injection of 6.0 × 10^5^ human CEnCs (n = 3; Group 2 control), and the other had the DM scraped similar to the treatment group, but were only exposed to an injection of solution containing ROCKi Y-27632 without CEnCs (n = 3; Group 3 control).

The concept of the delivery of CE-CI was based on previous reports^33,39,40^. Briefly, prior to CE-CI, a single intravenous dose of heparin (500 units in 1.0 ml) was administered to the rabbits to reduce intraocular fibrin formation. Subsequently, an anterior chamber maintainer was placed to infuse a balanced salt solution (BSS) containing additional heparin (1 unit per ml). Next, a paracentesis was created with a diamond knife to accommodate the insertion of a 30-gauge silicone soft tipped cannula (catalogue number: SP-125053, ASICO, Illinois, USA) (Supplementary Figure S4A) for the scrapping of the CEnCs, limbus to limbus, whilst keeping the DM intact (Supplementary Figure S4B). This was performed for both rabbits in Group 1 CE-CI and Group 3 control. Continuous irrigation with BSS ensured endothelial cells did not remain on the surface of the DM. A solution of trypan blue was injected intracamerally to aid in the assessment of the DM denudation (Supplementary Figure S4C). Areas of DM devoid of CEs were stained blue, and any areas with residual CE stood out against blue-stained DM (arrowed; Supplementary Figure S4D). The scraping process was then repeated to target these areas specifically until the entire DM was stained blue, indicating all corneal endothelial cells had been removed (Supplementary Figure S4E). Subsequently, 0.5 mL of 100 μg/mL carbochol (Miostat®, Alcon Laboartories) was injected to achieve intraoperative miosis. Both the paracentesis incision and the anterior chamber maintainer paracentesis sites were secured with 10/0 nylon interrupted sutures. This was followed by a 0.2 mL anti-inflammatory and anti-infective subconjunctival injection of a 1:1 mixture of 4 mg/mL dexamethasone sodium phosphate (Hospira, Melbourne, Australia) and 40 mg/mL gentamicin sulfate (Shin Poong Pharmaceutical, Seoul, Korea). Using a syringe and 30 G cannula, 0.4 ml of aqueous humour was removed to shallow the anterior chamber. Cultured CEnCs suspended in ROCKi Y-27632 and M5-endo medium were then injected through a separate tunneled track via a 30 G needle (Supplementary Figure S4F). Immediately following CE-CI, rabbits were placed in a manner that ensured the cornea was in a downward position; and maintained for three hours under volatile anesthesia.

#### Post-transplantation care

Following TE-EK or CE-CI, all rabbits received a post-operative regime of topical prednisolone acetate 1% (Allergan Inc, New Jersey, USA) and topical antibiotic tobramycin 1% (Alcon Laboratories) four times a day. An intramuscular injection of 1 mL/kg dexamethasone sodium phosphate (Norbrook Laboratories, Northern Ireland, UK) was also administered once daily. This medication regime was maintained until the rabbits were sacrificed.

### Corneal imaging and intra-ocular pressure measurement

All corneal imaging and measurements of intra-ocular pressure (IOP) were performed prior to transplantation, as well as at 4 days, 1, 2, and 3 weeks after surgical procedures. Slit lamp photographs were taken with a Zoom Slit Lamp NS-2D (Righton, Tokyo, Japan) and corneal cross-sectional scans and measurements of corneal thickness were performed using an anterior segment optical coherence tomography system (AS-OCT; Optovue, California, USA). Three measurements were taken for the assessment of central corneal thickness (CCT): at the corneal center (0.0 mm), and at 1 mm either side of the center (+1.0 mm, and −1.0 mm), and the mean value reported. Measurements of IOP were measured using a calibrated tonometer (Tono-pen Avia Vet, Reichert Ophthalmic Instruments, New York, USA). *In-vivo* confocal images were obtained using the Heidelberg Retina Tomography (HRT) 3 system combined with the Rostock Corneal Module (HRT3/RCM; Heidelberg Engineering, Heidelberg, Germany) to evaluate corneal endothelial cell density following TE-EK or CE-CI, where random areas of between 50 to 100 cells were assessed for cell density using the software within. A minimum of at least 3 confocal images was evaluated to obtain the corneal endothelial cell density.

### Analysis of corneas

All rabbits were followed for 21 days following surgery before being sacrificed under anesthesia with an overdose of intracardiac injection of 85 mg/kg sodium pentobarbitone (Jurox, New South Wales, Australia).

#### Immunohistochemistry

For immunohistochemistry, excised corneal samples were embedded in frozen section compounds (Surgipath; Leica Microsystems, Nussloch, Germany), and stored at −80 °C until sectioning. Serial sections of 10 µm sections were cut using a HM525 NX cryostat (Thermo Scientific) and collected on polylysin-coated glass slides (Thermo Scientific). Samples were rinsed and blocked in 5% normal goat serum in PBS for 30 min at room temperature. Subsequently, samples were incubated with the primary antibodies at room temperature for 1 hour or at 4 °C overnight. The primary antibodies used were Na^+^/K^+^-ATPase and ZO-1, as well as anti-human nuclei antibodies (Merck Millipore, Massachusetts, USA). Subsequently, samples were labeled with an AlexaFluor 488 conjugated goat anti-mouse IgG secondary antibody (2.5 µg/ml, Life Technology), mounted in Vectashield containing DAPI (Vector Laboratories, California, USA), and visualized using a Zeiss Axioplan 2 fluorescence microscope (Carl Zeiss, Oberkochen, Germany).

#### Histochemistry

For histochemistry, excised corneal sample was placed endothelial side up and stained for 3 minutes in a buffered trypan blue solution (0.2%), and subsequently stained in freshly prepared and filtered Alizarin red solution (0.5%; pH 4.5). The stained specimen was then washed for 60 seconds in a wash buffer, prior to wet mounting and examined using an Axioplan 2 microscope (Carl Zeiss AG, Oberkochen, Germany).

#### Scanning electron microscopy

Excised corneal specimens for scanning electron microscopy were first immersed overnight in a fixative solution consisting of 2% glutaraldehyde in PBS (pH 7.4; Electron Microscopy Sciences, Pennsylvania, USA) at 4 °C overnight. Fixed specimens were left in 3 washes of PBS for 5 minutes each, and kept in 1% osmium tetroxide at room temperature for 1 hour. The samples were then dehydrated in an increasing concentration of ethanol; 25%, 50%, 75%, 95% and 100% (10 minutes at each concentration), with the last step repeated 3 times. Dehydrated samples were then dried in a critical point dryer (BALTEC, Balzer, Liechtenstein), and mounted onto a SEM stub using carbon adhesive tabs. Samples were then sputter-coated with a 10 nm layer of gold (BALTEC), and examined under a scanning electron microscope (Quanta 650FEG; FEI, Oregon, USA).

### Statistical analysis

Data was managed in Excel (Microsoft) and analysed using Statistical Program for Social Sciences (SPSS©) Version 22 (IBM, New York, USA). Differences in the distribution of continuous variables between groups were analysed using the two-tailed independent t-test. When the distributions of more than two groups were compared, one-way analysis of variance (ANOVA) with Bonferroni correction was used. Significance level was set at *p* < 0.05.

## Supplementary information

Supplementary Information
