# Supplementary material for: Functional Evaluation of Two Corneal Endothelial Cell-Based Therapies: Tissue-Engineered Construct and Cell Injection
Source: Sci Rep. 2019 Apr 15;9:6087. doi: 10.1038/s41598-019-42493-3 (PMC6465252; doi:10.1038/s41598-019-42493-3)
Supplement: Supplementary file 1 — Supplementary Information [file 41598_2019_42493_MOESM1_ESM.docx]

Supplementary Information

Functional Evaluation of Two Corneal Endothelial Cell-Based Therapies: Tissue-Engineered Construct and Cell Injection

^†^*Gary S. L. Peh^1,2^*,* ^†^*Hon Shing Ong^1,3^, Khadijah Adnan^1^, Heng-Pei Ang^1^, Chan N. Lwin^1^, Xin-Yi Seah^1^, Shu-Jun Lin^1^, Jodhbir S. Mehta^1,2,3,4^**

^1^Tissue Engineering and Stem Cell Group, Singapore Eye Research Institute, Singapore.

^2^ Duke-NUS Graduate Medical School, Singapore.

^3^ Singapore National Eye Centre, Singapore.

^4^School of Material Science and Engineering, Nanyang Technological University, Singapore.

^†^These authors contributed equally to the writing of this manuscript.

* To whom correspondence should be addressed: garypeh@gmail.com / jodmehta@gmail.com

**
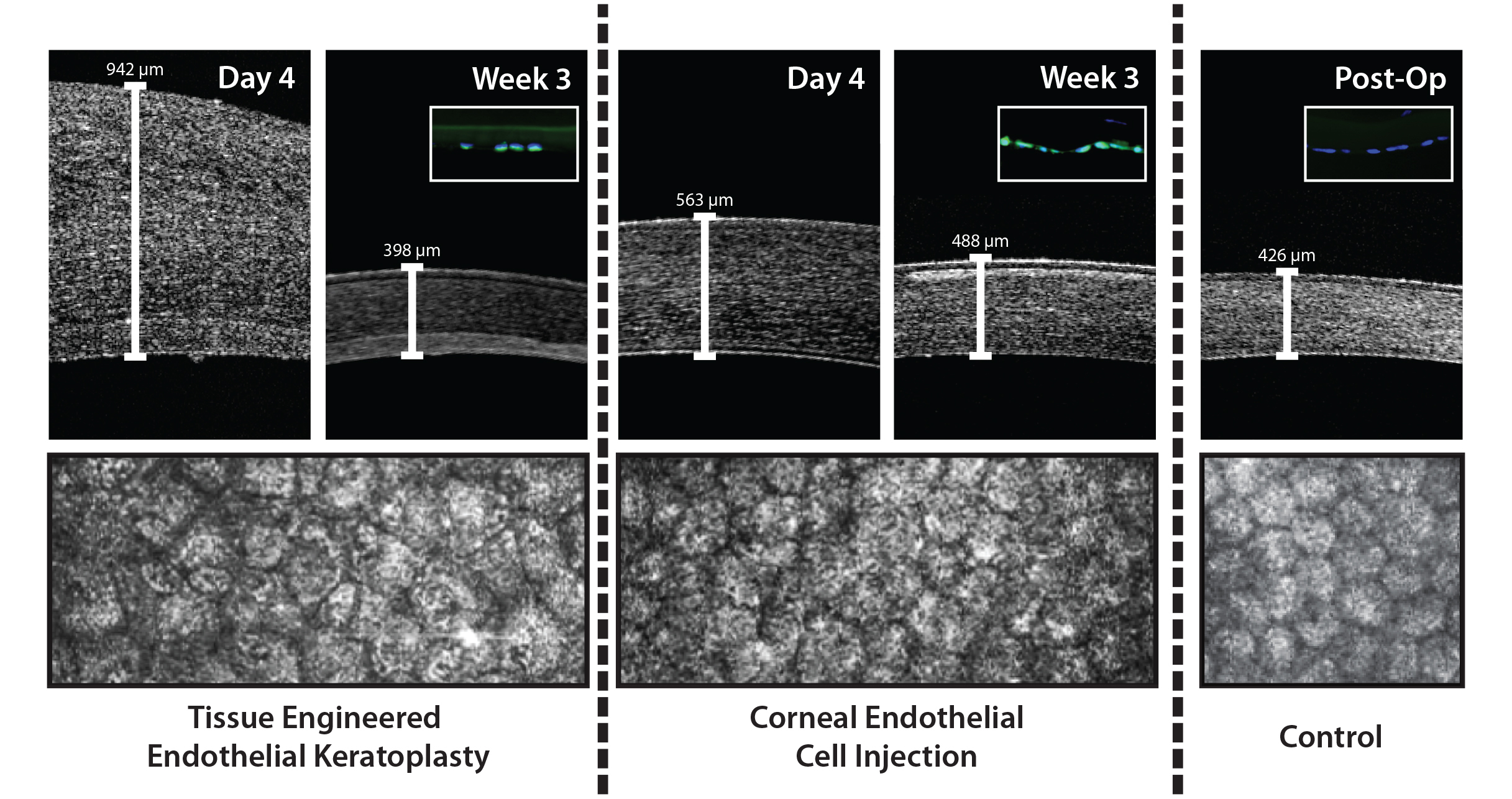
**

**Supplementary Figure S1 | Recovery, corneal endothelial cell density and morphometric analysis for TE-EK vs CE-CI.** This figure shows representative AS-OCT and *in vivo* confocal images of the corneas of rabbit receiving a TE-EK graft, and rabbit obtaining the CE-CI procedure at both Day 4 and at Week 3, as well as that of a control rabbit. Corneal endothelial cell density was taken at the third week, and showed a cell count of 1,248 ± 64 cells/mm^2^ for TE-EK and 1,469 ± 128 cells/mm^2^ for CE-CI. Cell circularity measurement was 0.86 ± 0.04 and 0.84 ± 0.06 for TE-EK and CE-CI respectively.

**
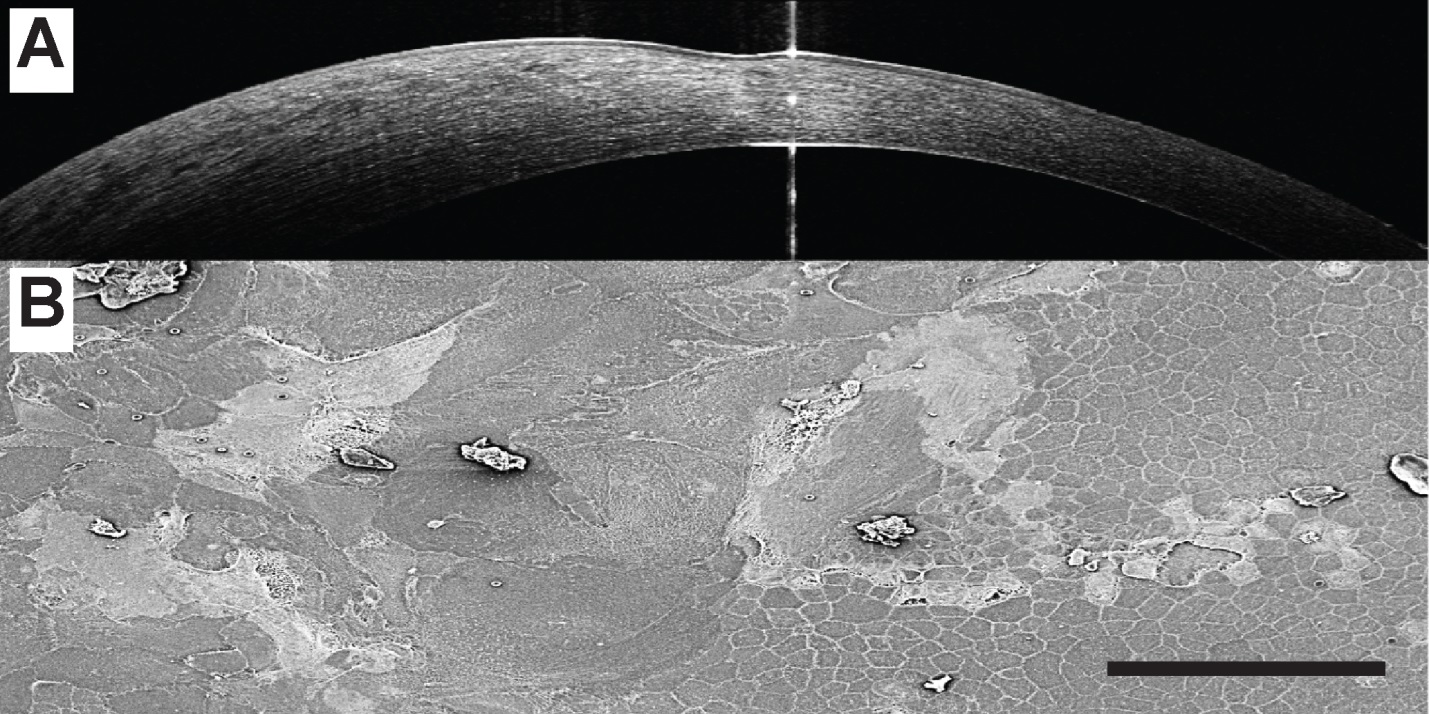
**

**Supplementary Figure S2 | Improper posturing for CE-CI.** Representative (A) AS-OCT image showed uneven thickness of the rabbit cornea and (B) is a corresponding SEM image where CEnCs were observed on the side of the cornea that was thinner. The side of the cornea that was thicker had no observable CEnCs. Scale bar: B 250 µm.

**
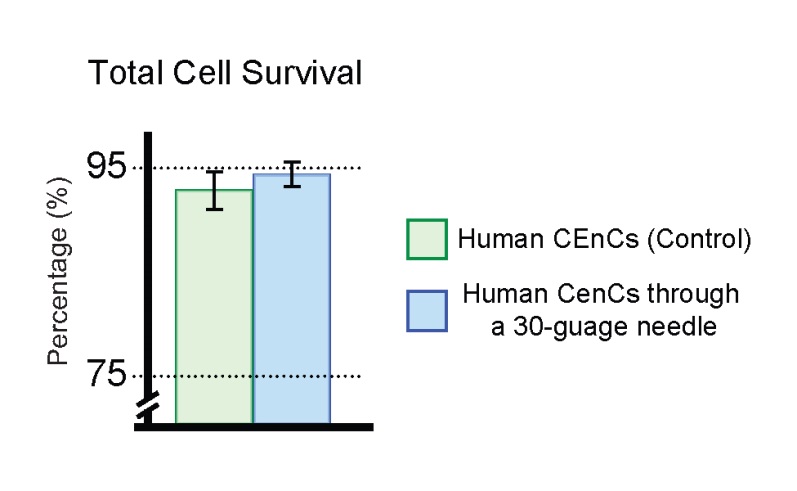
**

**Supplementary Figure S3 | Cell viability.** Cell viability of primary human CEnCs that were passed through a 30 gauge needle to simulate CE-CI were assessed for their viability by flow cytometry, and results showed comparative cellular survival to those of regular CEnCs control following cellular dissociation into single cells.

**
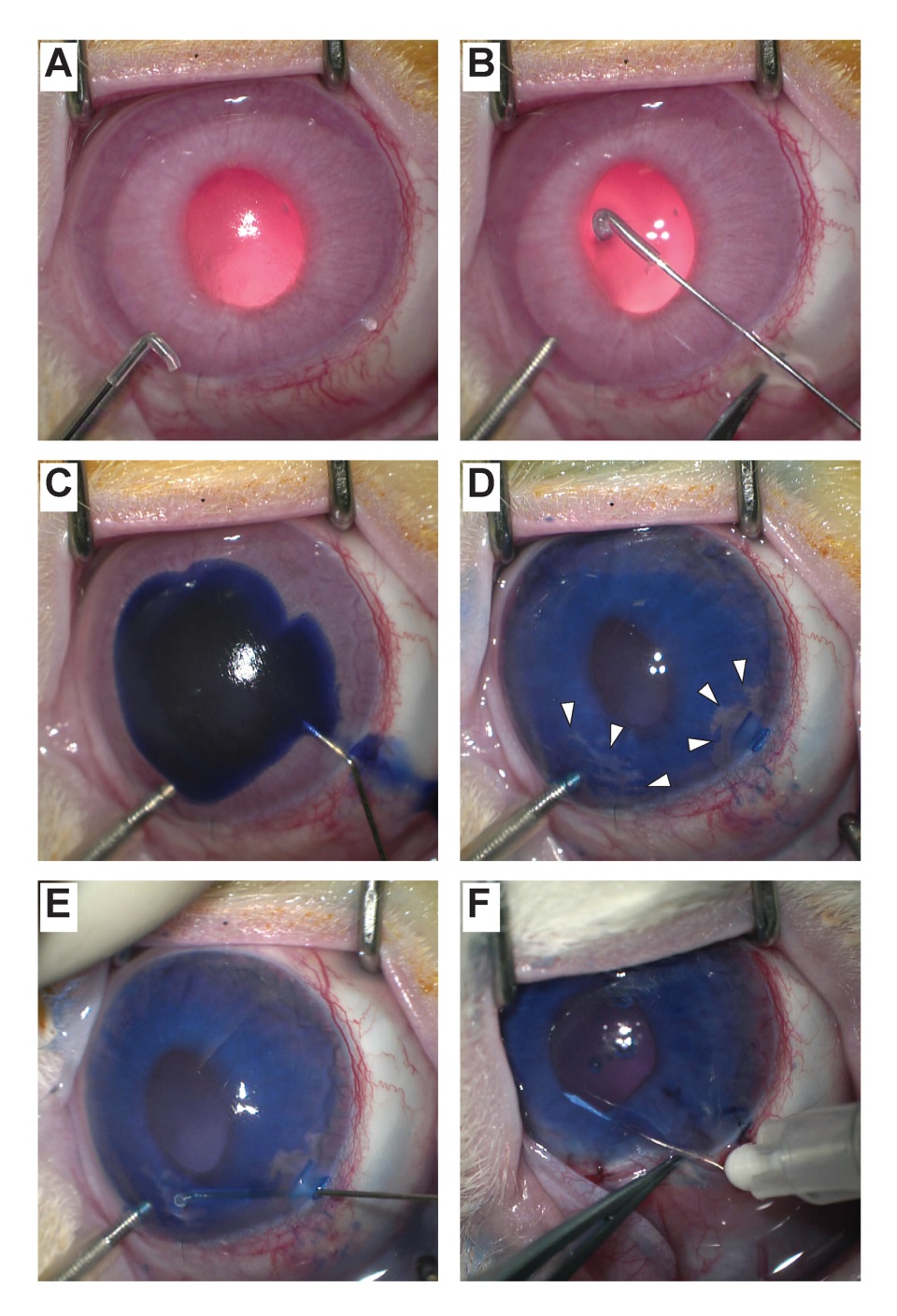
**

**Supplementary Figure S4 | Removal of rabbit’s naïve corneal endothelial cells.** (A) Representative images of the rabbit’s eye together with the 30-gauge silicone soft tipped cannula. (B) Scrapping of the rabbit’s corneal endothelium using the soft tipped cannula, keeping the DM intact. (C) Trypan blue staining of the anterior chamber to visualized any remaining corneal endothelial cells. (D) Remaining corneal endothelial cells (white arrows) as contrasted by trypan blue, which stained the DM, but not the cells. (E) Re-insertion of the soft tipped cannula into the anterior chamber to remove the remaining corneal endothelial cells. (F) Injection of cultured human CEnCs into the rabbit’s anterior chamber.
